# Coculture with monocytes/macrophages modulates osteogenic differentiation of adipose‐derived mesenchymal stromal cells on poly(lactic‐co‐glycolic) acid/polycaprolactone scaffolds

**DOI:** 10.1002/term.2826

**Published:** 2019-04-05

**Authors:** Hongbo Tang, Johanna F.A. Husch, Yang Zhang, John A. Jansen, Fang Yang, Jeroen J.J.P. van den Beucken

**Affiliations:** ^1^ Department of Biomaterials, Radboud Institute for Molecular Life Sciences Radboudumc Nijmegen The Netherlands; ^2^ Department of Plastic Surgery, Tongji Hospital, Tongji Medical College Huazhong University of Science and Technology Wuhan China

**Keywords:** 3D, adipose‐derived mesenchymal stromal cells, coculture, macrophages, monocytes, osteogenic differentiation

## Abstract

The effects of immune cells, in particular macrophages, on the behaviour of mesenchymal stromal cells (MSCs) have recently gained much attention for MSCs‐based tissue‐engineered constructs. This study aimed to evaluate the effect of monocytes/macrophages on the osteogenic differentiation of adipose‐derived mesenchymal stromal cells (ADMSCs) in three‐dimensional (3D) cocultures. For this, we cocultured THP‐1 monocytes, M1 macrophages, or M2 macrophages with ADMSCs on 3D poly(lactic‐co‐glycolic) acid (PLGA)/polycaprolactone (PCL) scaffolds using osteogenic medium for up to 42 days. We found that osteogenic differentiation of ADMSCs was inhibited by monocytes and both macrophage subtypes in 3D scaffolds. Furthermore, coculture of monocytes/macrophages with ADMSCs resulted in downregulated secretion of oncostatin M (OSM) and bone morphogenetic protein 2 (BMP‐2) and inhibited expression of osteogenic markers alkaline phosphatase (ALP), bone sialoprotein (BSP), and runt‐related transcription factor 2 (RUNX2). Compared with both macrophage subtypes, monocytes inhibited osteogenic differentiation of ADMSCs more significantly. These data suggest that the mutual interactions between monocytes/macrophages and ADMSCs negatively affect MSC osteogenic differentiation and thus possibly bone healing capacity, which highlights the importance of the micro‐environment in influencing cell‐based constructs to treat bone defects and the potential to improve their performance by resolving the inflammation ahead of treatment.

## 1 INTRODUCTION

Inflammation is the first stage of bone healing after bone injury. The state of inflammation has been indicated to affect the delicate balance between bone formation and bone degradation (Loi et al., 2016). Monocytes and macrophages are vital modulators of inflammation (Nich et al., 2013) and display the transition of different phases in tissue regeneration (Wynn & Vannella, 2016). The crosstalk between monocytes/macrophages and cells involved in tissue regeneration, such as mesenchymal stromal cells (MSCs), is critical for normal tissue formation and healing (Guihard et al., 2012; Guihard et al., 2015; Vi et al., 2015). Upon injury, monocytes are recruited from the peripheral circulation and enter injured sites, where they differentiate into macrophages (Rickard & Young, 2009). The recruited macrophages respond to signals from the micro‐environment in which they reside by acquiring different phenotypes (Wynn & Vannella, 2016). These macrophages are generally classified as either classically activated macrophages (M1) or alternatively activated macrophages (M2; Murray et al., 2014; Spiller, Freytes, & Vunjak‐Novakovic, 2015). Based on current knowledge, M1 macrophages are responsible for angiogenesis and the removal of necrotic tissue at an early stage, whereas M2 macrophages are responsible for immune regulation, matrix deposition, and tissue remodelling at a later stage (C. Chen, Uludag, Wang, Rezansoff, & Jiang, 2012). Recent studies reported a switch of macrophage subtypes from pro‐inflammatory M1 macrophages to pro‐wound healing M2 macrophages during the bone healing process (Tasso et al., 2013; Wu et al., 2015), demonstrating the crucial role of monocytes and different macrophage subtypes in bone healing.

To further elucidate the interaction of different macrophage subtypes with bone forming cells, such as MSCs, in vitro, our group has previously established a two‐dimensional (2D) coculture system where different types of macrophages were cocultured with adipose‐derived mesenchymal stromal cells (ADMSCs; Zhang et al., 2017). This study demonstrated that M2 macrophages, rather than M1 macrophages, can promote the osteogenic differentiation of ADMSCs. Although culturing cells on 2D substrates has been considered a standard technique for in vitro cell culture, it is recognized that cells more closely mimic native tissues when cultured in a three‐dimensional (3D) environment. In 3D cell cultures, cells adhere to each other via the extracellular matrix (ECM) and form specific cell–cell contacts, which differentially regulate cell growth, migration, and differentiation (Lee, Cuddihy, & Kotov, 2008). This is supported by findings of significant divergence of cell–cell interactions for cells in 2D and 3D culture systems in previous studies (D. Y. Chen et al., 2013; Valles et al., 2015). Furthermore, 3D scaffolds are widely used for tissue engineering applications. The most widely used materials for tissue engineering are polymeric materials because they are easily processable, biocompatible, and biodegradable and can be modified with desired properties (e.g., dimensions and porosity; Ceccarelli et al., 2013). In recent years, polymers have been processed via electrospinning to fabricate nanofibres for different applications in skin (Duan et al., 2006), blood vessel (Vaz, van Tuijl, Bouten, & Baaijens, 2005), and bone tissue regeneration (Zhang et al., 2008). Electrospun fibres represent morphological similarity to natural ECM, which makes them attractive for cells to proliferate and function effectively (Yang, Yang, Wang, Both, & Jansen, 2013). The interfibre pores that are obtained within electrospun fibre meshes render such scaffolds highly interactive with its surrounding tissue due to the high specific surface area (Holzwarth & Ma, 2011). To make full use of the functionality of multiple polymer types in one electrospun mesh, the blend electrospun method, which allows the simultaneous combination of multiple polymers during the electrospinning process, has gained much attention (Hiep & Lee, 2010). An attractive polymer combination for electrospun meshes includes poly(lactic‐co‐glycolic) acid (PLGA), which is suitable for cell adhesion and proliferation due to its hydrophilic properties, and polycaprolactone (PCL), which is a flexible biopolymer that can be used to overcome the brittle and low elongation properties of PLGA (Kim & Cho, 2009).

For initial biological evaluation using cell culture models, culture conditions for cocultures require special attention regarding medium composition and nutritional supplement. Although standardized culture conditions have been established for most monocultures (American Type Culture Collection, 2018), coculture models require a justified choice for a specific medium. Mostly, this choice is based on the research question that favours behavioural analysis of the predominant cell type within the coculture, for example, vascular cells for angiogenic behaviour (Hofmann et al., 2008; Levenberg et al., 2005) and MSCs for osteogenic differentiation (Ma et al., 2011; Ma et al., 2014). As a nutritional supplement, fetal bovine serum (FBS) has been commonly used for multiple cell types. However, the major drawback of this supplement is the possibility to trigger an immunological response due to the presence of xenogeneic antigens (Bieback et al., 2009). Consequently, it has been postulated that the use of FBS should be avoided for human cell cultures (Ma et al., 2015). In contrast, platelet lysate (PL) is of human origin, can be applied as an autologous nutritional supplement for primary cells, and contains various growth factors and cytokines, including platelet‐derived growth factor (PDGF), basic fibroblast growth factor (bFGF), insulin‐like growth factor 1 (IGF‐1), and transforming growth factor β (TGF‐β; Doucet et al., 2005). A vast amount of scientific literature has reported on the capacity of PL to promote the proliferation and differentiation of MSCs into different lineages (Altaie, Owston, & Jones, 2016; Fekete et al., 2012; Shanskii et al., 2013). In particular, PL has been demonstrated to be an optimal serum supplement to culture ADMSCs for bone regeneration (Hayrapetyan, Bongio, Leeuwenburgh, Jansen, & van den Beucken, 2016; Ma et al., 2015). Furthermore, PL was also used in tissue‐engineered scaffolds to benefit the innate immune response for superior tissue regeneration. It was found that PL can induce an anti‐inflammatory response of monocytes/macrophages (Linke et al., 2017). These findings suggest the potential of using PL to culture human cells for the clinical usage.

The objective of this study was to evaluate the effect of monocytes and macrophage subtypes on osteogenic differentiation of ADMSCs cultured on 3D PLGA/PCL scaffolds using a direct coculture model. We hypothesized that monocytes and macrophage subtypes would differentially affect the osteogenic differentiation of ADMSCs compared with ADMSCs monocultures on PLGA/PCL scaffolds.

## 2 MATERIALS AND METHODS

### 2.1 Cells and reagents

ADMSCs were obtained from human subcutaneous adipose tissue, and human monocytic THP‐1 cells were obtained from the American Type Culture Collection (Manassas, VA, USA). Alpha Minimum Essential Medium (αMEM), RPMI‐1640 medium, and penicillin–streptomycin were purchased from Gibco (GrandIsland, USA). FBS, bovine serum albumin (BSA), trypsin, bFGF, phorbol‐12‐myristate‐13‐acetate (PMA), lipopolysaccharide (LPS), interferon gamma (IFN‐γ), interleukin 4 (IL‐4), IL‐13, β‐glycerol 2‐phosphate disodium salt hydrate (β‐glycerophosphate), dexamethasone, and ascorbic acid were purchased from Sigma‐Aldrich (St. Louis, USA). Heparin was obtained from LEO Pharma A/S (Ballerup, Denmark). Collagenase A was purchased from Roche Diagnostics (Mannheim, Germany). Tumour necrosis factor alpha (TNF‐α) and TGF‐β enzyme‐linked immunosorbent assay (ELISA) kits were purchased from eBioscience (San Diego, USA). Oncostatin M (OSM) and bone morphogenetic protein 2 (BMP‐2) ELISA kits were purchased from Sigma‐Aldrich (St. Louis, USA). Monoclonal anti‐human CCR7 antibody was purchased from Abcam (Cambridge, UK), mouse purified anti‐human CD36 was obtained from BioLegend (San Diego, USA), and mouse anti‐human CD68 was purchased from Dako (Heverlee, Belgium). All secondary antibodies and 4′,6‐diamidino‐2‐phenylindole (DAPI) were purchased from Invitrogen (Waltham, USA). All cell culture flasks, dishes, and plates were purchased from Greiner Bio‐One (Frickenhausen, Germany).

### 2.2 Isolation, preculture, and characterization of ADMSCs

ADMSCs isolation was performed as described previously (Varma et al., 2007). Briefly, human subcutaneous adipose tissue was obtained from the Department of Plastic Surgery (Radboudumc, Nijmegen, the Netherlands) after ethical approval (CMO Radboudumc; dossier# 2017‐3252) and written informed consent. Resected fat tissue was minced using surgical scalpels and scissors and washed with phosphate‐buffered saline (PBS) for three times. The tissue was digested with 0.1% collagenase A in PBS containing 1% BSA at 37°C for 60 min with intermittent shaking. The digested tissue was centrifuged for 10 min at 600 g, and the cell pellet was resuspended in 5 ml of PBS/1% BSA and filtered with a 100‐μm nylon mesh (Roche Diagnostics). Cells were then subjected to a Ficoll density centrifugation (Lymphoprep™, 1,000 g, 20 min; Axis‐Shield, Oslo, Norway) step to remove erythrocytes and were seeded at a density of 1 × 10^5^ cells/cm^2^ in αMEM containing 10% FBS, 100‐U/ml penicillin, 100‐μg/ml streptomycin, and 1‐ng/ml bFGF, and cultured in a humidified atmosphere of 5% CO_2_ at 37°C. Medium was changed twice a week. When near confluent (90%), cells were detached with 0.5‐mM EDTA/0.05% trypsin and passaged or frozen in 1 × 10^6^ cells/ml aliquots in liquid nitrogen. ADMSCs from passages 3 to 5 were used in further experiments.

The expression of surface antigens was evaluated by incubating ADMSCs at 4°C for 1 hr with the respective antibodies in 100‐μl FACS buffer (1‐mM EDTA in PBS with 0.5% BSA; Sigma). The following antibodies were used for evaluation: FITC mouse anti‐human CD45, APC mouse anti‐human CD73, PerCP‐Cy 5.5 mouse anti‐human CD90, and PE mouse anti‐human CD105 (all from BD Pharmingen, Piscataway, USA). Cells without antibodies were used as negative controls. Labelled cells were washed twice in 1‐ml FACS buffer and analysed with the FACSAria II flow cytometer (BD biosciences, San Jose, CA, USA). Data were processed using Flowing software 2.5.1 (University of Turku, Turku, Finland), and the percentage population of each antibody that stained positively for the respective markers was compared with negative controls.

### 2.3 Culture, activation, and polarization of THP‐1 cells

Human monocytic THP‐1 cells were cultured in RPMI‐1640 medium supplemented with 10% FBS, 100‐U/ml penicillin, and 100‐μg/ml streptomycin. THP‐1 cells were differentiated into macrophages using a previously published protocol (Freytes, Kang, Marcos‐Campos, & Vunjak‐Novakovic, 2013). Briefly, 5 × 10^6^ cells were added into 100‐mm culture dishes with 15‐ml culture medium plus 50‐ng/ml PMA for 48 hr to activate monocytes into M0 macrophages. Then M0 macrophages were treated for another 48 hr either with 20‐ng/ml IFN‐γ and 240‐ng/ml LPS to obtain M1 macrophages, or with 20‐ng/ml IL‐4 and 20‐ng/ml IL‐13 to obtain M2 macrophages. After the polarization, the supernatant was collected for characterization, and the cells were washed twice with PBS. The cells were then used for coculture with ADMSCs.

M1 and M2 phenotypes were characterized by measuring the concentration of TNF‐α and TGF‐β in collected supernatants from polarized macrophages using the respective ELISA kits. ELISA kits were used according to the manufacturer's instructions. M1 and M2 phenotypes were further characterized by immunostaining for the M1 macrophage marker CCR7 and M2 macrophage marker CD36 (Stewart, Yang, Makowski, & Troester, 2012). The cells were fixed with 4% paraformaldehyde for 15 min and then blocked with incubation buffer (1% BSA in PBS) for 1 hr at room temperature. Samples were incubated with the primary antibodies rabbit anti‐human CCR7 (1:500) and mouse anti‐human CD36 (1:100) for 2 hr in incubation buffer. After washing three times with PBS, the cells were incubated with the secondary antibodies donkey anti‐rabbit Alexa Fluor 568‐labelled IgG and goat anti‐mouse Alexa Fluor 488‐labelled IgG (both 1:200) for 1 hr at room temperature in the dark. After washing three times with PBS, the cells were incubated with DAPI for 5 min. Immunofluorescence images were acquired with a fluorescence microscope (Zeiss AxioCam MRc5, Carl Zeiss Microimaging GmbH, Germany), and the relative intensity of fluorescence was analysed using ImageJ (U.S. National Institutes of Health, Bethesda, USA). The value of red (Alexa 568) and green (Alexa 488) fluorescence of each sample was further normalized for the value of blue fluorescence (DAPI), as described previously (Zhang et al., 2017).

### 2.4 Preparation of PL

PL was prepared as described previously (Prins et al., 2009). Briefly, pooled platelet products containing approximately 1 × 10^9^ thrombocytes/ml were purchased from the Sanquin Blood Bank (Nijmegen, the Netherlands). The product was divided into 5‐ml aliquots in 15‐ml tubes (Greiner Bio‐One), subjected to one freeze/thaw (−80°C/37°C) cycle and stored at −80°C until use. Before adding to the medium, PL was thawed and centrifuged at 2,000 *g* for 10 min to remove remaining platelet fragments.

### 2.5 Scaffolds preparation and cell loading

PLGA (Purasorb® PDLG 8531, Purac Biomaterials BV, Gorinchem, the Netherlands) and PCL (LACTEL® Absorbable Polymers, DURECT Corporation, Cupertino, CA, USA) were used in the electrospinning process. Organic solvent 2,2,2‐trifluoroethanol (purity 99.8%) was obtained from Acros (Geel, Belgium). The electrospinning solution was prepared by dissolving PLGA/PCL (weight ratio 3:1) in 2,2,2‐trifluoroethanol at a concentration of 0.12 g/ml.

The 3D scaffolds were fabricated using a so‐called wet‐electrospinning technique in a commercially available electrospinning set‐up (Esprayer ES‐2000S, Fuence, Tokyo, Japan). The optimal processing parameters for stable formation of electrospun fibres were selected based on an earlier publication (Yang et al., 2013). Briefly, the prepared polymer solution was fed into a glass syringe and delivered to an 18G nozzle at a feeding rate of 50 μl/min. A high voltage (20–25 kV) was applied at the nozzle to generate a stable polymer jet by overcoming the surface tension of the polymer solution. A grounded bath filled with 100% ethanol located 15 cm under the nozzle was used to collect the fibres. To control the size of resulting fibre meshes, the process was stopped every 15 min for fibre mesh collection. Subsequently, the wet‐electrospun scaffolds were washed thoroughly in MilliQ and freeze‐dried (VirTis BenchTop Pro with Omnitronics Freeze Dryer, SP Scientific, NY, USA) for 3 days. The obtained scaffold displayed an uncompressed structure with an average fibre diameter of 1.98 ± 0.51 μm and a porosity of 99% (Yang et al., 2013).

Disk‐shaped scaffolds with a diameter of 6 mm and a thickness of about 2 mm were punched out using a biopsy punch (Kai medical, Gifu, Japan) from each wet‐electrospun mesh and subsequently sterilized in 70% ethanol for 2 hr and soaked in proliferation medium overnight.

**Figure 1:**
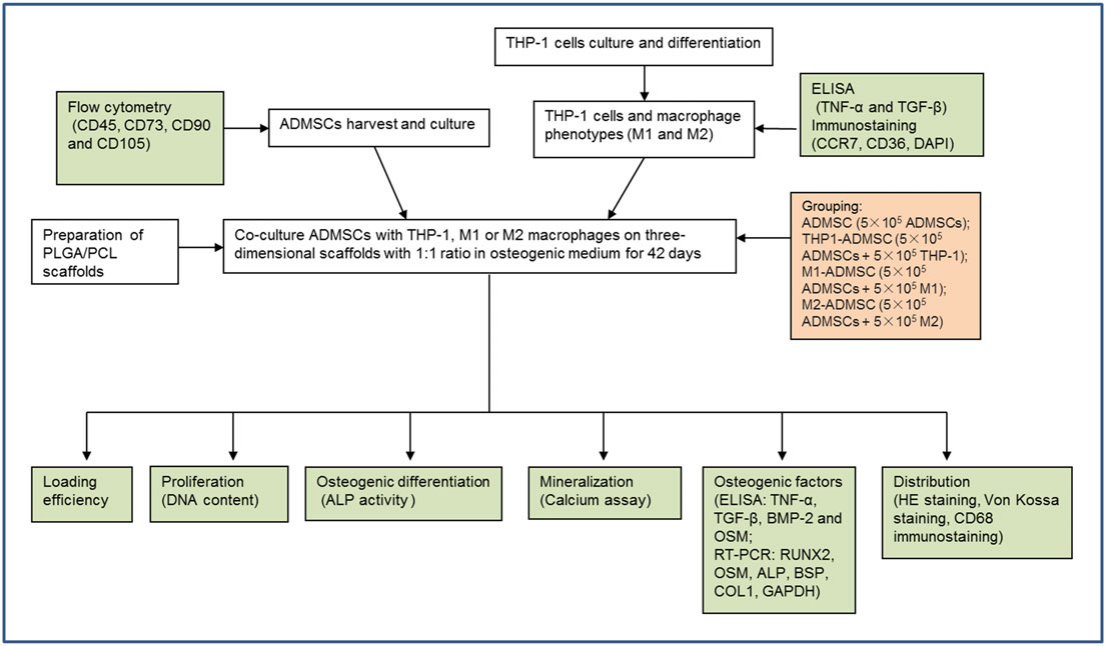
Schematic representation of the experimental design. ADMSCs: adipose‐derived mesenchymal stromal cells; ALP: alkaline phosphatase; TNF‐α: tumour necrosis factor alpha; BMP‐2: bone morphogenetic protein 2; BSP: bone sialoprotein; COL1: collagen type 1; DAPI: 4′,6‐diamidino‐2‐phenylindole; GAPDH: glyceraldehyde 3‐phosphate dehydrogenase; HE: haematoxylin and eosin; OSM: oncostatin M; PCL: polycaprolactone; PLGA: poly(lactic‐co‐glycolic) acid; RT‐PCR: reverse transcription polymerase chain reaction; RUNX2: runt‐related transcription factor 2; TGF‐β: transforming growth factor β [Colour figure can be viewed at wileyonlinelibrary.com]

The experimental procedure of this study is schematically represented in Figure 1. To analyse the distinctive roles of monocytic THP‐1 cells, M1 macrophages, and M2 macrophages on the osteogenic differentiation of ADMSCs, four experimental groups were used:

1. ADMSC (5 × 10^5^ ADMSCs; monoculture control)
2. THP1‐ADMSC (5 × 10^5^ ADMSCs with 5 × 10^5^ THP‐1 cells)
3. M1‐ADMSC (5 × 10^5^ ADMSCs with 5 × 10^5^ M1 macrophages)
4. M2‐ADMSC (5 × 10^5^ ADMSCs with 5 × 10^5^ M2 macrophages)

The scaffolds were placed into 96‐well plates. The cells were trypsinized and resuspended in proliferation medium, and 5 × 10^5^ cells in 25‐μl medium were seeded onto each scaffold. Scaffolds were incubated for 3 hr for initial attachment, and then 300‐μl proliferation medium was added; 24 hr later, the scaffolds were placed in 48‐well suspension plates, and 1‐ml osteogenic medium (αMEM supplemented with 5% PL, 10‐U/ml heparin, 1% penicillin–streptomycin antibiotic mixture, 50‐μg/ml ascorbic acid, 10‐mM β‐glycerophosphate, and 10^−8^‐M dexamethasone) was added. The cell–scaffold constructs were cultured for 42 days, and the medium was refreshed thrice a week.

### 2.6 Analyses

#### 2.6.1 Cell loading efficiency and DNA content

To evaluate the cell loading efficiency for each group, DNA content of the loaded cells was measured after 24 hr of seeding. Scaffolds with cells were also collected on Days 3, 7, 14, and 28 for DNA content measurement (*n* = 3). Samples were washed twice with PBS, transferred to 1.5‐ml Eppendorf tubes, and digested with 0.1% collagenase A in PBS and 1% BSA for 16 hr at 37°C with intermittent shaking. The digested samples were centrifuged for 5 min at 2,000 *g*. The supernatant was aspirated, and 1 ml of MilliQ was added to each tube after which repetitive freezing (−80°C) and thawing (room temperature) cycles were performed; 5 × 10^5^ ADMSCs with or without 5 × 10^5^ of another type of cells were suspended in 1 ml of MilliQ, which was regarded as 100% control. DNA content was quantified using the QuantiFluor dsDNA System (Promega Corporation, Madison, USA) according to the manufacturer's instructions. A DNA standard curve was used to quantify the amount of DNA in each sample, and the results were measured using a multimode microplate reader (Synergy HTX, Bio‐Tek Instruments, Vermont, USA) with an excitation wavelength at 485/20 nm and an emission wavelength at 528/20 nm. Loading efficiency in each group was calculated through division of the result by the respective 100% control.

#### 2.6.2 ALP activity

Gross alkaline phosphatase (ALP) activity was measured using the same samples as described for the DNA content measurement. For the assay, 80 μl of sample and 20 μl of buffer solution (0.5‐M AMP) were added in 96‐well plates. Then 100 μl of substrate solution (5‐nM *p*‐nitrophenylphosphate disodium salt; Sigma‐Aldrich, St. Louis, USA) was added in all the wells, and the plates were incubated at 37°C for 1 hr. The reaction was stopped by adding 100 μl of stop solution (0.3‐M NaOH; Sigma‐Aldrich, St. Louis, USA). For the standard curve, serial dilutions of 4‐nitrophenol were added to a final amount of 0–25 nmol. The absorbance of the samples was read using a multimode microplate reader (Synergy HTX, Bio‐Tek Instruments, Vermont, USA) at 405 nm.

#### 2.6.3 Mineralization

Mineralization was measured using a calcium assay (orthocresolphtalein complexone; Sigma). Samples were collected on Days 14, 28, and 42. Scaffolds were washed twice with PBS, after which 1 ml of 0.5‐N acetic acid (Sigma‐Aldrich, the Netherlands) was added to each well (*n* = 3). The plate was incubated on a shaking table overnight at room temperature. For the assay, 10 μl of sample or standard was pipetted in a 96‐well plate, followed by the addition of 300‐μl working solution. Working solution consisted of five portions of 14.8‐M ethanolamine/boric acid buffer (pH = 11), five portions of orthocresolphtalein complexone solution, two portions of 8‐hydroxyquinoline, and 88 portions of MilliQ. For the standard curve, serial dilutions of calcium stock (CaCl_2_) were prepared to final concentrations of 0–100 μg/ml. The plates were incubated at room temperature for 5–10 min, and the absorbance was read using a multimode microplate reader (Synergy HTX, Bio‐Tek Instruments, Vermont, USA) at 570 nm.

#### 2.6.4 Cytokine secretion analysis by ELISA

After 3, 7, 14, 28, and 42 days in culture, the culture medium was collected, centrifuged, and stored at −20°C until analyses were performed (*n* = 3). The concentrations of BMP‐2, OSM, TNF‐α, and TGF‐β were determined using ELISA kits according to the manufacturer's instructions. Culture medium with cell‐free scaffolds served as blanks.

#### 2.6.5 RNA extraction and real‐time qPCR

Gene expression was studied after 3, 7, 14, and 28 days of osteogenic differentiation (*n* = 4). Briefly, scaffolds with cells were washed with PBS and cut into small pieces before adding 1 ml of Trizol (Invitrogen, Breda, the Netherlands). The cell extract was collected, mixed with chloroform (Sigma‐Aldrich, St. Louis, USA), and centrifuged. Only the upper aqueous phase was collected and mixed with equal amount of isopropanol (Sigma‐Aldrich, St. Louis, USA). After 10 min of incubation at room temperature, the mixture was centrifuged and washed with 75% alcohol. Thereafter, the obtained RNA pellet was dissolved in RNase‐free water, and the RNA concentration was measured with a spectrophotometer (NanoDrop 2000, Thermo Scientific, Wilmington, DE, USA).

First‐strand cDNA was reverse transcribed from RNA using the iScript™ Select cDNA Synthesis Kit (Bio‐Rad, California, USA). Afterwards, cDNA was further amplified, and the expression of specific genes was quantified using quantitative polymerase chain reaction (qPCR) MasterMix Plus for SYBR® Green I (Eurogentec, Seraing, Belgium) and a real‐time PCR detection system (CFX96™ Real‐Time PCR Detection system, Bio‐Rad). Osteogenic differentiation‐related marker genes were evaluated, including ALP, bone sialoprotein (BSP), collagen type 1 (COL1), runt‐related transcription factor 2 (RUNX2), and osteocalcin (OCN). The sequence of applied primers is given in Table 1. The expression levels were analysed and compared with the housekeeping gene glyceraldehyde 3‐phosphate dehydrogenase (GAPDH). The specificity of the primers was confirmed separately before the real‐time PCR reaction. The expression of the tested genes was calculated using the 2^−∆∆Ct^ method (Schmittgen & Livak, 2008) using ADMSC group on Day 3 as the reference group.

**Table 1:** Primer sequences used for real‐time quantitative polymerase chain reaction

Gene	Forward (5′ → 3′)	Reverse (5′ → 3′)
ALP	CCCAAAGGCTTCTTCTTG	CTGGTAGTTGTTGTGAGCAT
BSP	AACCTACAACCCCACCACAA	AGGTTCCCCGTTCTCACTTT
COL1	GGTGTAAGCGGTGGTGGTTAT	GCTGGGATGTTTTCAGGTTGG
OCN	GACTGTGACGAGTTGGCTGA	CTGGAGAGGAGCAGAACTGG
RUNX2	GGAGTGGACGAGGCAAGAGTTT	AGCTTCTGTCTGTGCCTTCTGG
GAPDH	CTCTGCTCCTCCTGTTCGACA	ACGACCAAATCCGTTGACTC

*Note*. ALP: alkaline phosphatase; BSP: bone sialoprotein; COL1: collagen type 1; GAPDH: glyceraldehyde 3‐phosphate dehydrogenase; OCN: osteocalcin; RUNX2: runt‐related transcription factor 2.

#### 2.6.6 Histological staining and immunohistochemistry

To visualize the distribution of cells in the scaffolds, samples were collected on Days 14, 28, and 42 (*n* = 3) and then fixed in 10% phosphate‐buffered formalin for 24 hr and dehydrated through graded ethanol, cleared with xylene, and embedded in paraffin. Serial sections (thickness 5 μm) were cut using a microtome (Leica RM2165, Nussloch, Germany) from each sample and used for haematoxylin and eosin (HE), CD68, and Von Kossa staining.

For CD68 staining, samples were put in sodium citrate (Sigma‐Aldrich, Zwijndrecht, the Netherlands) and heated to 70°C for 10 min in microwave oven. After rinsing in PBS, samples were preincubated with 10% donkey serum (Sigma‐Aldrich, Zwijndrecht, the Netherlands) for 10 min and then incubated with the primary antibody mouse anti‐human CD68 clone KP1 (1:200; Dako, Glostrup, Denmark) overnight at 4°C. After rinsing in PBS, samples were incubated with the biotinylated secondary antibody donkey anti‐mouse (1:500; Merck, Darmstadt, Germany) for 60 min and counterstained with haematoxylin (Sigma‐Aldrich, Zwijndrecht, the Netherlands) for 10 s. Samples were then dehydrated through a graded series of ethanol and mounted.

For Von Kossa staining (only on Day 42), 5% silver nitrate (Sigma‐Aldrich, Zwijndrecht, the Netherlands) was added, and samples were placed under ultraviolet light for 30 min. After rinsing in MilliQ, 2% sodium thiosulfate (Merck, Darmstadt, Germany) was added to remove the unreacted silver for 5 min. Next, samples were washed with running tap water for 10 min and counterstained with nuclear fast red (Polysciences, Warrington, PA, USA) for 10 min. Samples were then dehydrated through a graded series of ethanol and mounted.

### 2.7 Statistics

Data are presented as mean ± standard deviation (*SD*). Statistical analysis was performed by SPSS Statistics 19 software (IBM, New York, USA). Quantitative results were analysed using one‐way analysis of variance followed by Fisher's least significance difference test. *p* values less than 0.05 were considered statistically significant.

## 3 RESULTS

### 3.1 Characterization of ADMSCs

**Figure 2:**
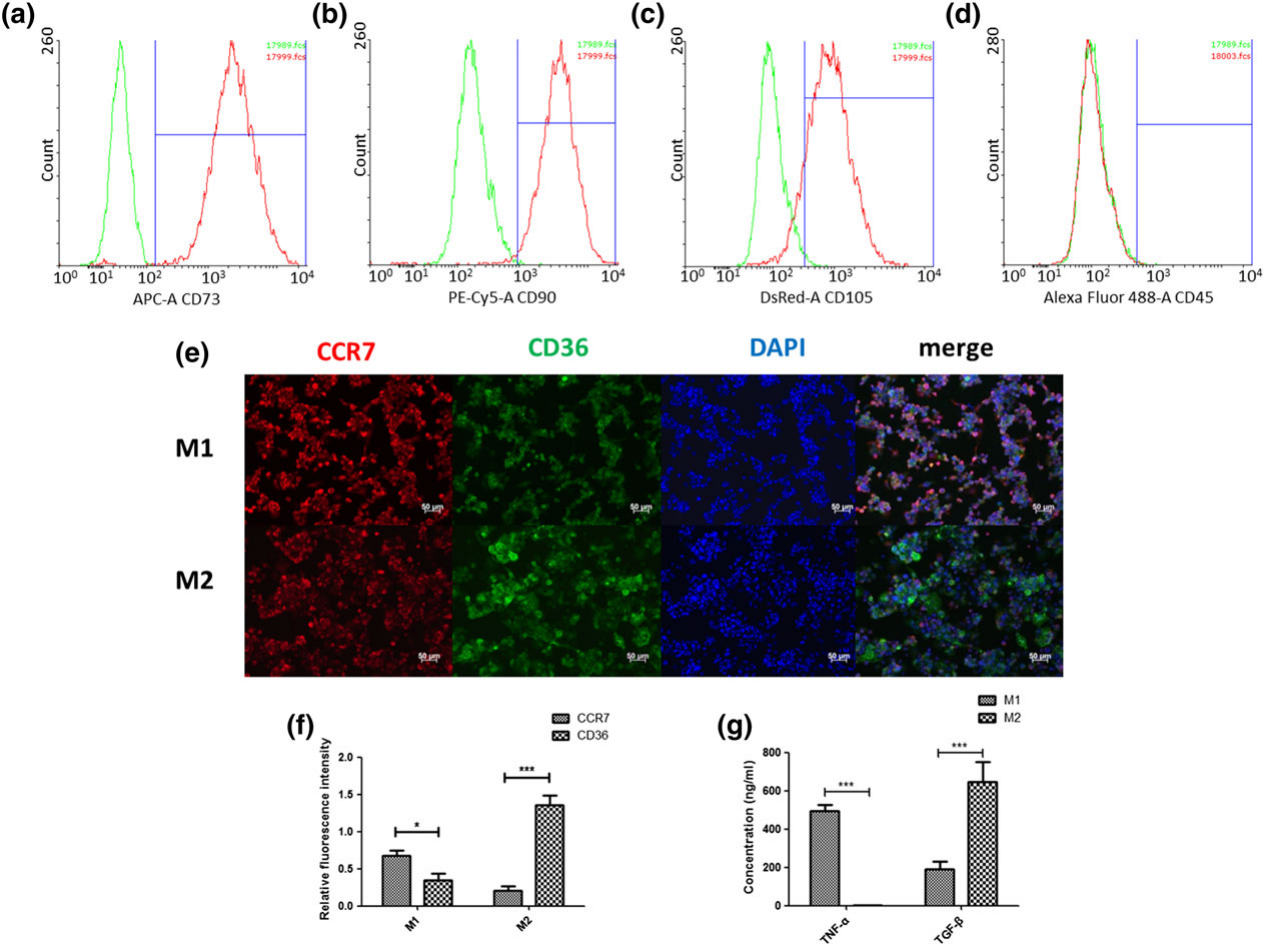
Characterization of adipose‐derived mesenchymal stromal cells (ADMSCs), M1, and M2 macrophages. (a–d) The ratio of positive cells in ADMSCs was compared with the ratio in negative controls. Gates were set using the negative control. Red lines indicate the histogram for ADMSCs markers, and green lines indicate the histogram for negative controls. (e) M1 macrophages and M2 macrophages were stained with M1 marker CCR7 (red), M2marker CD36 (green), and 4′,6‐diamidino‐2‐phenylindole (DAPI) (blue). (f) Quantification of relative fluorescence intensity of CCR7 and CD36 in M1 macrophages and M2 macrophages. (g) The concentration of tumour necrosis factor alpha (TNF‐α) and transforming growth factor β (TGF‐β) in M1 and M2 macrophage culture medium was measured by ELISA. Scale bar, 50 μm. “*” indicates significant difference between groups. *p < 0.05, **p < 0.01, ***p < 0.001 [Colour figure can be viewed at wileyonlinelibrary.com]

ADMSCs showed positive expression for MSC surface markers CD73 (99.4 ± 0.2%), CD90 (98.0 ± 1.4%), and CD105 (86.1 ± 3.5%) and negative expression of CD45 (0.24 ± 0.0%; Figure 2a–d). Evaluation of mineralization proved the osteogenic differentiation capacity of primary human ADMSCs with calcium content values for ADMSCs monocultures on Days 14 and 28 of 64.6 ± 7.4 and 313.0 ± 28.0 μg/ml, respectively (data not shown).

### 3.2 Characterization of M1 and M2 macrophages

Human THP‐1 monocytic cells were induced into M0 macrophages via activation with PMA and further differentiated into M1 or M2 macrophages using LPS/IFN‐γ or IL‐4/IL‐13, respectively. Activation with PMA changed the THP‐1 cells from cells growing in suspension to adherent cells. Morphologically, M1 macrophages showed a more spindle‐like shape compared with M2 macrophages (data not shown). Cytokine secretion levels of TNF‐α and TGF‐β were measured to analyse induction of M1 and M2 macrophages (Figure 2g). A significantly higher TNF‐α concentration was measured for M1 macrophages (492.8 ± 33.6 ng/ml) compared with M2 macrophages (3.1 ± 0.7 ng/ml; *p* < 0.001). In contrast, a significantly higher concentration of TGF‐β was determined for M2 macrophages (647.7 ± 103.5 ng/ml) compared with M1 macrophages (188.7 ± 43.0 ng/ml; *p* < 0.001).

Immunostaining for macrophage phenotypes showed a mixture of M1 and M2 macrophages after induction (Figure 2e). Following polarization into M1 macrophages, more positive staining for the M1 marker CCR7 and less positive staining for the M2 marker CD36 were observed. In contrast, M2 polarized macrophages demonstrated increased positive staining for CD36 and less positive staining for CCR7. Upon fluorescence signal quantification, significant differences between M1 and M2 macrophages were determined (Figure 2f). M1 macrophages showed a significantly higher expression of CCR7 (0.679 ± 0.127; *p* < 0.05) than CD36 (0.351 ± 0.178), the opposite was observed for M2 macrophages, with CD36 (1.359 ± 0.247; *p* < 0.001) being significantly higher expressed compared with CCR7 (0.209 ± 0.109).

### 3.3 Cell seeding efficiency and DNA content

**Figure 3:**
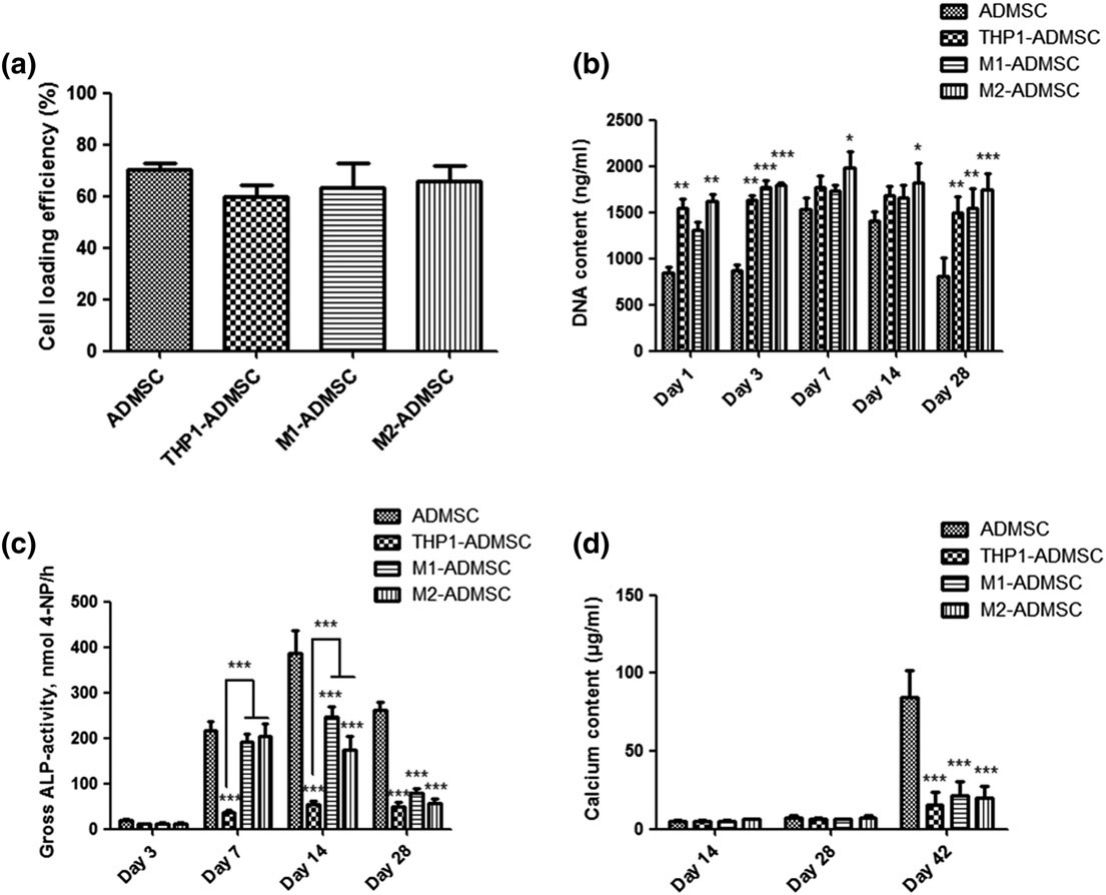
(a) Cell seeding efficiency in each group. (b) DNA content in each group on Days 1, 3, 7, 14, and 28. (c) Gross alkaline phosphatase (ALP) activity in each group on Days 3, 7, 14, and 28. (d) Calcium content in each group on Days 14, 28, and 42. “*” indicates significant difference compared with the adipose‐derived mesenchymal stromal cell (ADMSC) group at the same time point. *p < 0.05, **p < 0.01, ***p < 0.001

Twenty‐four hours after cell seeding, the DNA content of the adherent cells for each experimental group was measured to assess the cell seeding efficiency (Figure 3a). Similar cell seeding efficiencies of 70.4 ± 4.0%, 59.6 ± 8.6%, 63.4 ± 15.9%, and 66.0 ± 9.9% were observed for the ADMSCs monoculture, THP1‐ADMSCs, M1‐ADMSCs, and M2‐ADMSCs cocultures, respectively (*p* > 0.05).

All experimental groups showed a slight increase in DNA content from Days 1 to 7 and then a slight decrease from Days 14 to 28 (Figure 3b). On Days 1 and 3, the DNA content of the ADMSCs monoculture was significantly lower compared with all other groups (*p* < 0.01). On Days 7 and 14, the DNA content of the monoculture was significantly lower compared with the M2‐ADMSCs coculture (*p* < 0.05). On Day 28, the DNA content of the ADMSCs monoculture was significantly lower compared with all other groups (*p* < 0.01). At each time point, similar DNA content values were measured for all coculture groups (*p* > 0.05).

### 3.4 ALP activity

ADMSCs monoculture and M1‐ADMSCs and M2‐ADMSCs cocultures showed a similar trend for ALP activity over the culture period, that is, a rise during the early stage and a decline in the later stage (Figure 3c). On both Days 14 and 28, the gross ALP activity for ADMSCs was significantly higher compared with all other experimental groups (*p* < 0.001). For the THP1‐ADMSCs coculture, a lower gross ALP activity was observed throughout the entire culture. On both Days 7 and 14, the gross ALP activity for the THP1‐ADMSCs coculture was significantly lower compared with all other coculture groups (*p* < 0.001).

### 3.5 Mineralization

Mineralization levels for all experimental groups were relatively low on Days 14 and 28, without significant differences among groups (*p* > 0.05; Figure 3d). On Day 42, the calcium content for the ADMSCs monoculture was significantly higher compared with all other groups (*p* < 0.001).

### 3.6 Cytokine secretion analysis by ELISA

**Figure 4:**
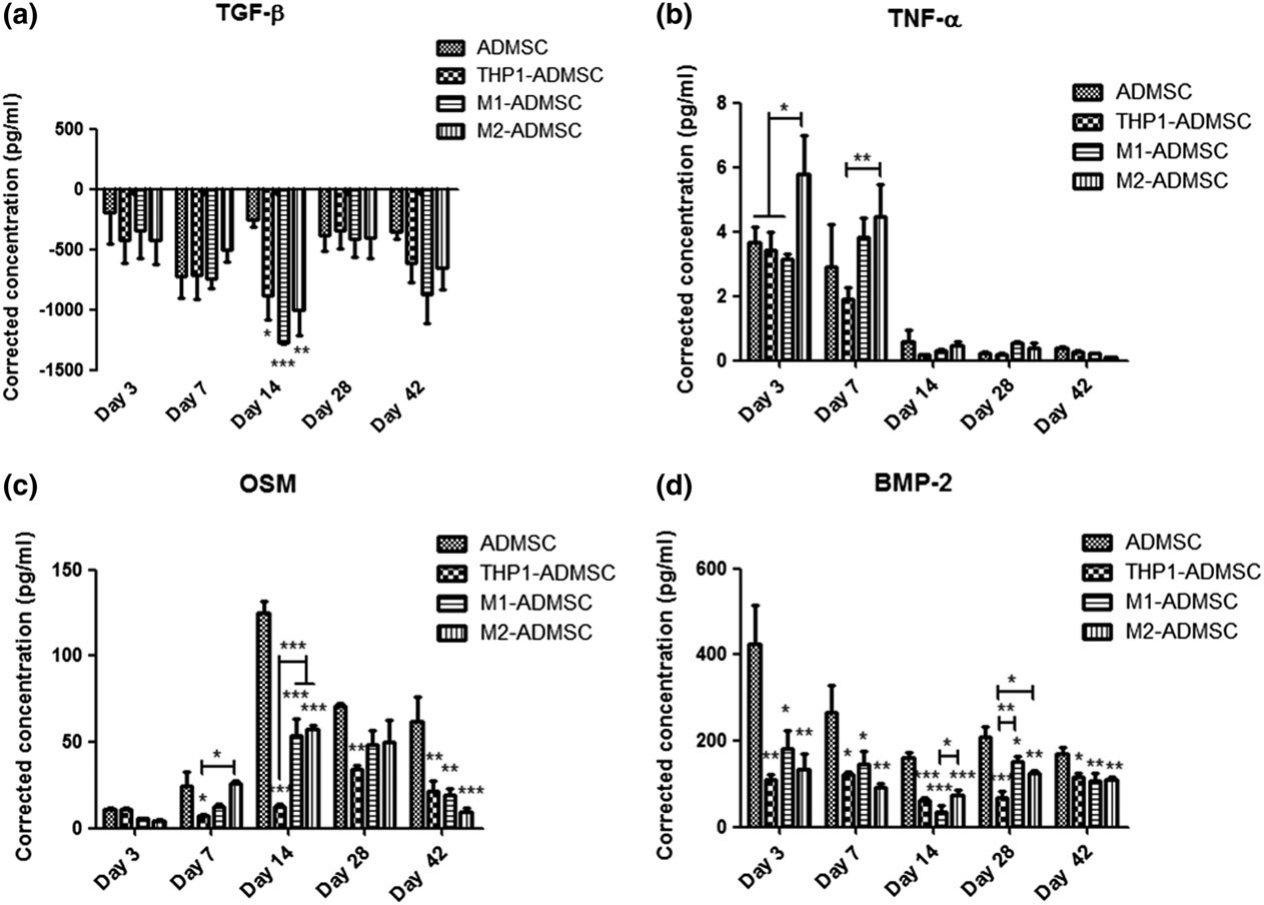
Cytokine secretion analysis by ELISA. (a) Corrected transforming growth factor β (TGF‐β) concentration in each group on Days 3, 7, 14, 28, and 42. (b) Corrected tumour necrosis factor alpha (TNF‐α) concentration in each group on Days 3, 7, 14, 28, and 42. (c) Corrected oncostatin M (OSM) concentration in each group on Days 3, 7, 14, 28, and 42. (d) Corrected bone morphogenetic protein 2 (BMP‐2) concentration in each group on Days 3, 7, 14, 28, and 42. “*” indicates significant difference compared with the adipose‐derived mesenchymal stromal cell (ADMSC) group at the same time point. *p < 0.05, **p < 0.01, ***p < 0.001

After correction by blank values, TGF‐β concentrations showed negative values at all time points. On Day 14, the TGF‐β concentration of coculture groups was significantly lower compared with the ADMSCs monoculture (*p* < 0.05; Figure 4a).

TNF‐α concentration remained at a low level at all time points. On Day 3, TNF‐α concentration of the M2‐ADMSCs coculture was higher compared with the ADMSCs monoculture and the THP1‐ADMSCs coculture (*p* < 0.05; Figure 4b). On Day 7, the TNF‐α concentration of the M2‐ADMSCs coculture was higher compared with the THP1‐ADMSCs coculture (*p* < 0.01; Figure 4b).

OSM concentration showed an apparent trend of increasing at an early stage and decreasing at a late stage during culture for all experimental groups. On Days 14 and 42, OSM concentration of the ADMSCs monoculture was higher compared with all coculture groups (*p* < 0.001; Figure 4c).

The BMP‐2 concentration decreased with culture time. The BMP‐2 concentration of the ADMSCs monoculture was higher compared with all coculture groups at all time points (*p* < 0.05; Figure 4d).

### 3.7 Real‐time qPCR

**Figure 5:**
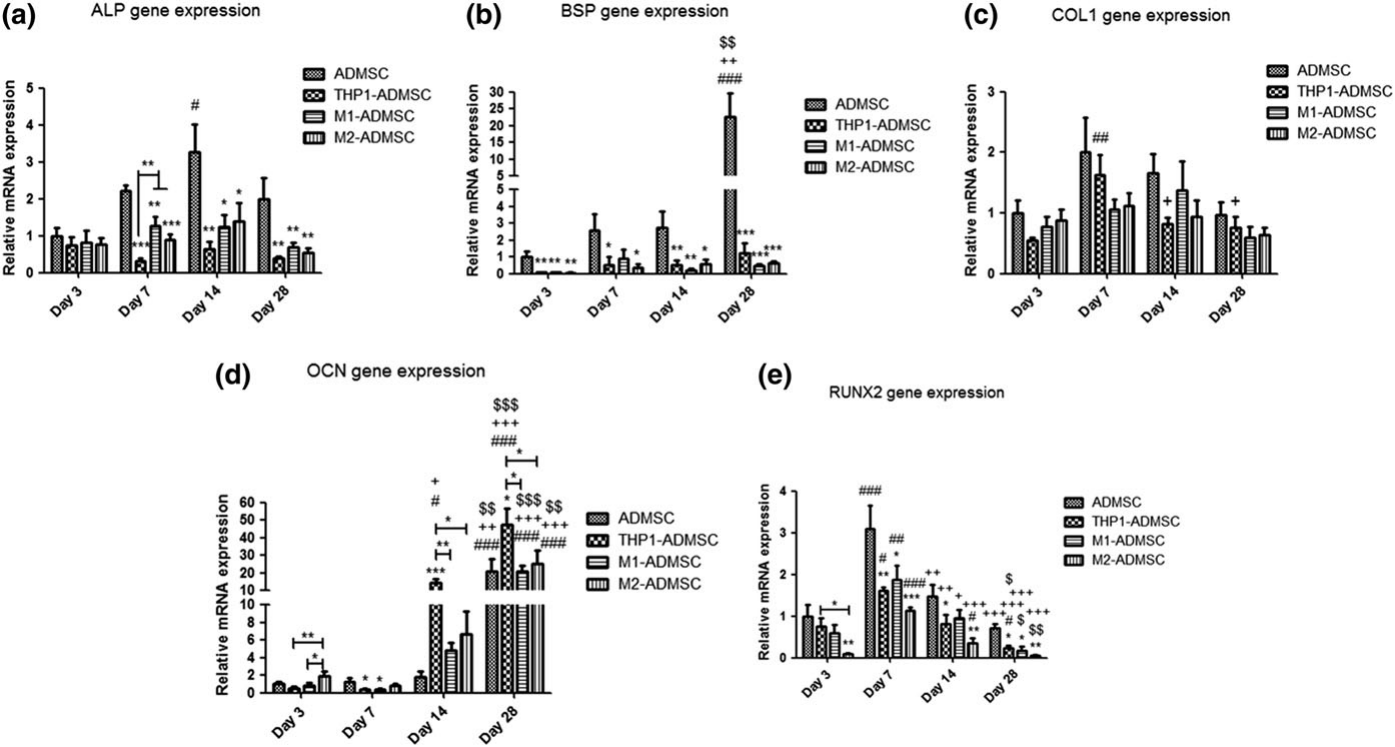
Gene expression of osteogenic markers. (a) Gene expression of alkaline phosphatase (ALP) in each group on Days 3, 7, 14, and 28. (b) Gene expression of bone sialoprotein (BSP) in each group on Days 3, 7, 14, and 28. (c) Gene expression of collagen type 1 (COL1) in each group on Days 3, 7, 14, and 28. (d) Gene expression of osteocalcin (OCN) in each group on Days 3, 7, 14, and 28. (e) Gene expression of runt‐related transcription factor 2 (RUNX2) in each group on Days 3, 7, 14, and 28. “*” indicates significant difference compared with the adipose‐derived mesenchymal stromal cell (ADMSC) group at the same time point. *p < 0.05, **p < 0.01, ***p < 0.001. “#” indicates significant difference compared with Day 3 in the same group. #*p* < 0.05, ##*p* < 0.01, ###*p* < 0.001. “+” indicates significant difference compared with Day 7 in the same group. +*p* < 0.05, ++*p* < 0.01, +++*p* < 0.001. “$” indicates significant difference compared with Day 14 in the same group. $*p* < 0.05, $$*p* < 0.01, $$$*p* < 0.001

Osteogenic differentiation of ADMSCs was inhibited upon coculture with monocytes or macrophage subtypes along with the decrease in the expression of osteogenesis‐related genes, which was examined by real‐time PCR analysis (Figure 5). For ALP gene expression, no significant differences among the experimental groups at Day 3 were observed (Figure 5a). Significantly, higher ALP gene expression was observed for the ADMSCs monoculture compared with the three coculture groups at Days 7 (*p* < 0.001), 14 (*p* < 0.05), and 28 (*p* < 0.01). For BSP gene expression, significantly higher expression was observed for the ADMSCs monoculture compared with the three coculture groups at Days 3 (*p* < 0.01), 7 (*p* < 0.05), 14 (*p* < 0.01), and 28 (*p* < 0.001; Figure 5b). No significant differences between ADMSC and the coculture groups were observed for COL1 gene expression at any time point (Figure 5c). For OCN gene expression, significantly higher expression was observed for the THP1‐ADMSCs coculture compared with the ADMSCs monoculture at Days 14 (*p* < 0.001) and 28 (*p* < 0.05; Figure 5d). Significantly higher expression was observed for THP1‐ADMSCs coculture compared with M1‐ADMSCs and M2‐ADMSCs cocultures at Days 14 and 28 (*p* < 0.05). For RUNX2 gene expression, significantly higher expression was observed for the ADMSCs monoculture compared with the M2‐ADMSCs coculture at each time point (*p* < 0.01; Figure 5e). Significantly higher expression was observed for ADMSCs monoculture compared with the THP1‐ADMSCs and M1‐ADMSCs cocultures at Days 7 (*p* < 0.01), 14 (*p* < 0.05), and 28 (*p* < 0.05).

### 3.8 Histological staining and immunohistochemistry

HE‐stained histological sections of all experimental groups are presented in Figure S1. For all experimental groups, the distribution of cells in the scaffolds was not homogeneous. At Day 14, most cells were distributed on the surface of the scaffolds, and only a small number of cells were observed in the centre of the scaffolds. From Day 14 to Days 28 and 42, increasingly more cells were observed infiltrating the scaffolds. A large number of cells mounted layer upon layer on the surface of the scaffolds, especially for the THP1‐ADMSCs, M1‐ADMSCs, and M2‐ADMSCs coculture groups.

The pan‐macrophage marker CD68 was used to monitor the distribution of monocytes/macrophages within coculture groups (Figure S2). No CD68‐positive staining was observed for the ADMSCs monoculture. In the coculture groups, CD68‐positive stained cells were mostly distributed on the superficial zone of the scaffolds. From Days 14 to 42, more and more monocytes/macrophages were observed infiltrating the scaffolds. No apparent differences in the distribution of CD68‐positive stained cells in all three coculture groups were observed.

Von Kossa staining was used to monitor mineral deposition (specifically PO_4_^3−^) within the scaffolds (Figure S3). No mineral deposition was observed for coculture groups over the entire culture period. However, the ADMSCs monoculture showed superficial mineralization on Day 42.

## 4 DISCUSSION

In view of the eminent role of monocytes and macrophages in the inflammatory cascade that initiates wound healing and tissue regeneration (Soltan, Rohrer, & Prasad, 2012), the objective of this study was to evaluate the effect of monocytes and macrophage subtypes on osteogenic differentiation of ADMSCs. Here, we used 3D PLGA/PCL scaffolds and a direct coculture model to culture primary human ADMSCs with THP1 monocytes, M1 macrophages, or M2 macrophages in osteogenic medium with a monoculture of ADMSCs serving as control. Our findings indicate that the osteogenic differentiation of ADMSCs is inhibited by monocytes and different macrophage subtypes in 3D scaffolds. Whereas low mineralization was observed for any of the cocultures, ADMSCs monoculture showed significantly higher mineralization after 42 days of culture. Further, cocultured ADMSCs with monocytes/macrophages showed a downregulation of the expression of osteogenic markers (e.g., ALP, BSP, and RUNX2) compared with ADMSCs monocultures, which are speculated to be related to OSM and BMP‐2 secretion of ADMSCs.

Regarding interaction between MSCs and monocytes/macrophages, several previous studies cocultured macrophages with MSCs and reported diverse effects (i.e., stimulatory or inhibitory) on the osteogenic differentiation of MSCs (C. Chen et al., 2012; Z. Chen et al., 2014; Fernandes et al., 2013; Guihard et al., 2012). This variation can be attributed to multiple factors, including the source of stem cells, utilized polarization protocols for macrophages, and cell ratios. Therefore, the exact role of monocytes/macrophages on osteogenic differentiation of MSCs requires a more comprehensive and more accurate research set‐up. Our group has developed a delicate, indirect 2D coculture system using transwells and the THP‐1 cell line as monocyte source and showed that different types of macrophages differentially affected the behaviour of cocultured ADMSCs (Zhang et al., 2017). To be more specific, M2 macrophages, rather than M1 macrophages, promoted the mineralization of ADMSCs and proved that this is mediated through paracrine signalling pathways. However, given the cell behavioural difference in 3D and 2D culture systems and the fact that 3D scaffolds are a crucial part of cell‐based bone constructs, we here established a direct 3D coculture system by using human primary ADMSCs, THP‐1 cells and electrospun scaffolds. In contrast to the previously observed stimulatory effects of macrophages on the osteogenic differentiation of ADMSCs (Zhang et al., 2017), monocytes, M1 macrophages, and M2 macrophages significantly inhibited the osteogenic differentiation of cocultured ADMSCs. This is evidenced by decreased ALP activity, mineralization content, and expression of several osteogenic markers. These results further validated previous reports that cell–cell interactions are different in 2D and 3D culture models (D. Y. Chen et al., 2013; Valles et al., 2015).

With regard to the mechanism behind this inhibitory effect of monocytes and macrophages, pro‐inflammatory and anti‐inflammatory cytokines were measured during the culture time, but no significant differences in inflammatory cytokine concentrations in the coculture medium were detected. However, a significantly decreased OSM and BMP‐2 secretion was found in cocultures compared with the monoculture. This indicated that monocytes/macrophages inhibited certain osteogenic signalling pathways in our 3D coculture system. The observed inhibitory effect may come from other cytokines than inflammatory cytokines such as TNF‐α and TGF‐β secreted by monocytes/macrophages (C. Chen et al., 2012) or the direct interaction between monocytes/macrophages, scaffold, and ADMSCs.

The other main difference of this 3D coculture compared with our previous 2D coculture model is the usage of PL instead of FBS as serum supplement for the sake of optimizing for potential clinical application. A rapidly increasing number of studies use PL, rather than FBS, as nutritional supplement in cell culture media for human cell culture (Astori et al., 2016; Burnouf, Strunk, Koh, & Schallmoser, 2016; Ruggiu, Ulivi, Sanguineti, Cancedda, & Descalzi, 2013). Except for the beneficial effects on the mineralization of ADMSCs, platelet derivatives were also shown to exert an anti‐inflammatory effect on monocytes/macrophages (Linke et al., 2017; Papait, Cancedda, Mastrogiacomo, & Poggi, 2018; Renn, Kao, Wang, & Burnouf, 2015). Furthermore, PL contains a high concentration of TGF‐β as reported previously, with fluctuations from 900 to 15,000 pg/ml (Fekete et al., 2012; Renn et al., 2015; Salvade et al., 2010). In our research, the concentration of TGF‐β in blank controls (i.e., culture medium incubated with scaffold only) was 2,426.6 ± 185.9 pg/ml (data not shown). These high values led to negative values after correction for blank controls. This observation suggests that the cells consume a large amount of TGF‐β in the process of proliferation and differentiation. Alternatively, the low concentrations of TNF‐α might imply the transition of M1 macrophages into M2 macrophages after 3 days of coculture with ADMSCs (Yin, Pang, Bai, Zhang, & Geng, 2016), which is a contradictory finding to an earlier publication suggesting that PL‐treated MSCs support the maintenance of macrophages in a pro‐inflammatory condition (Ulivi, Tasso, Cancedda, & Descalzi, 2014). However, the possible transition towards M2 macrophages might also explain the similarities regarding the osteogenic differentiation of ADMSCs upon coculture with either M1 or M2 macrophages in this study.

Macrophage differentiation and polarization are highly dynamic. In response to micro‐environmental cues, macrophages can rapidly switch from one phenotype to the other during the culture period (den Breems & Eftimie, 2016; Wang, Liang, & Zen, 2014). Generally, CCR7, HLA‐DR, CD163, and CD206 are used as markers for M1 or M2 macrophages. Nevertheless, none of these are fully discriminative for a particular subtype of macrophages (Spiller et al., 2014). Under these circumstances, we chose CD68 as a pan‐macrophage marker to assess the distribution of seeded monocytes or macrophages within coculture scaffolds. Combined with the results of HE, CD68, and Von Kossa staining, the two types of cells were uniformly distributed in the scaffolds. Most of the cells were distributed in a superficial layer of the scaffolds. Cells were mounted layer upon layer on the surface of the scaffolds. During the culture, ECM was deposited on the surface of the scaffolds forming a biofilm‐like structure. The migration of cells from the scaffold surface to the scaffold interior was likely hindered by this biofilm, which acted as a barrier (Lyons et al., 2010). This phenomenon might explain the low calcium content values and positive Von Kossa staining only at the surface of the scaffolds.

Molecular analysis of osteogenic markers showed that coculturing ADMSCs with monocytes or macrophages affect cytokine secretion (e.g., OSM and BMP‐2) and osteogenic gene expression (e.g., ALP, BSP, RUNX2, and OCN) of ADMSCs. Monocytes, M1 macrophages, and M2 macrophages significantly inhibited the osteogenic differentiation of cocultured ADMSCs in the early and late stages of osteogenesis, evidenced by lower ALP activity and BMP‐2 concentrations and lower gene expression of the early‐stage osteogenic marker ALP on Days 7 and 14 and late‐stage osteogenic marker BSP on Day 28. According to the comparison between coculture groups, monocytes played a stronger inhibiting role on ALP gene expression compared with M1 and M2 macrophages. Moreover, protein levels measured by ELISA showed that monocytes, M1 macrophages, and M2 macrophages significantly inhibited the secretion of OSM and BMP‐2 compared with ADMSCs monoculture. An interesting finding was the significantly increased OCN gene expression on Days 14 and 28 for the THP1‐ADMSCs coculture compared with all other experimental groups.

There are also several limitations to our study. First, to address multivariate research questions that require large numbers of cells and for reproducibility of results, the THP‐1 cell line rather than primary human monocytes and macrophages was used. Although THP‐1 cells have been reported to retain all necessary markers and morphologic features of primary monocytes (Auwerx, 1991; Qin, 2012), further studies using macrophages derived from primary monocytes of different donors are desired. Second, due to the lack of exclusive markers for M1 and M2 macrophages, we cannot dynamically monitor the macrophage behaviour during the culture time. A delicate staining method to follow the fate of macrophages and ADMSCs and to explore the cell–cell interaction would greatly aid in elucidating the mechanism of the observed inhibitory effects of monocytes/macrophages in vitro.

In conclusion, this study used cocultures of monocytes/macrophages and ADMSCs on 3D PLGA/PCL scaffolds to evaluate effects of cell–cell interactions on the osteogenic differentiation of ADMSCs. We found that monocytes and macrophage subtypes inhibit the osteogenic differentiation of ADMSCs on 3D PLGA/PCL scaffolds. Cocultured monocytes/macrophages decreased the expression of osteogenic markers ALP, BSP, and RUNX2. These data highlight the ignored fact that inflammation may regulate osteoblast activity of MSC‐based bone constructs within the bone micro‐environment. It implies that strict control of inflammation may be necessary to create an anabolic environment that improves the performance of cell‐based bone constructs. Additionally, compared with macrophage subtypes, monocytes played a stronger inhibiting role on the osteogenic differentiation of ADMSCs. Therefore, it seems that the transient activation of monocytes after fracture injury is important for fracture repair.

## CONFLICT OF INTEREST

The authors have declared that there is no conflict of interest.

## Supporting information


**Figure S1**. HE‐staining of paraffin sections for each experimental group after 14, 28 and 42 days of culture. Most cells (shown by arrows head) were distributed on the surface of the scaffolds at day 14. More cells were observed entering the scaffolds from day 14 to day 42. A large number of cells mounted layer upon layer on the surface of the scaffolds. “*” indicates scaffolds. Scale bar, 50μm.

**Figure S2**. CD68 staining of different group of PLGA/PCL scaffolds after 14, 28 and 42 days of culture. CD68‐positive cells stained in brown (shown by arrows head). No CD68‐positive staining was observed for the ADMSCs mono‐culture. “*” indicates scaffolds. Scale bar, 50μm.

**Figure S3**. Von Kossa staining of different group of PLGA/PCL scaffolds after 42 days of culture. Von Kossa staining was used to monitor mineral deposition (specifically PO_4_^3‐^) within the scaffolds. Mineral deposition stained in black (shown by arrows head). ADMSCs mono‐culture showed superficial mineralization at day 42. “*” indicates scaffolds. Scale bar, 100μm.
