# Supplementary figures and images for: Coculture with monocytes/macrophages modulates osteogenic differentiation of adipose‐derived mesenchymal stromal cells on poly(lactic‐co‐glycolic) acid/polycaprolactone scaffolds
Source: J Tissue Eng Regen Med. 2019 Apr 5;13(5):785–98. doi: 10.1002/term.2826 (PMC6594112; doi:10.1002/term.2826)

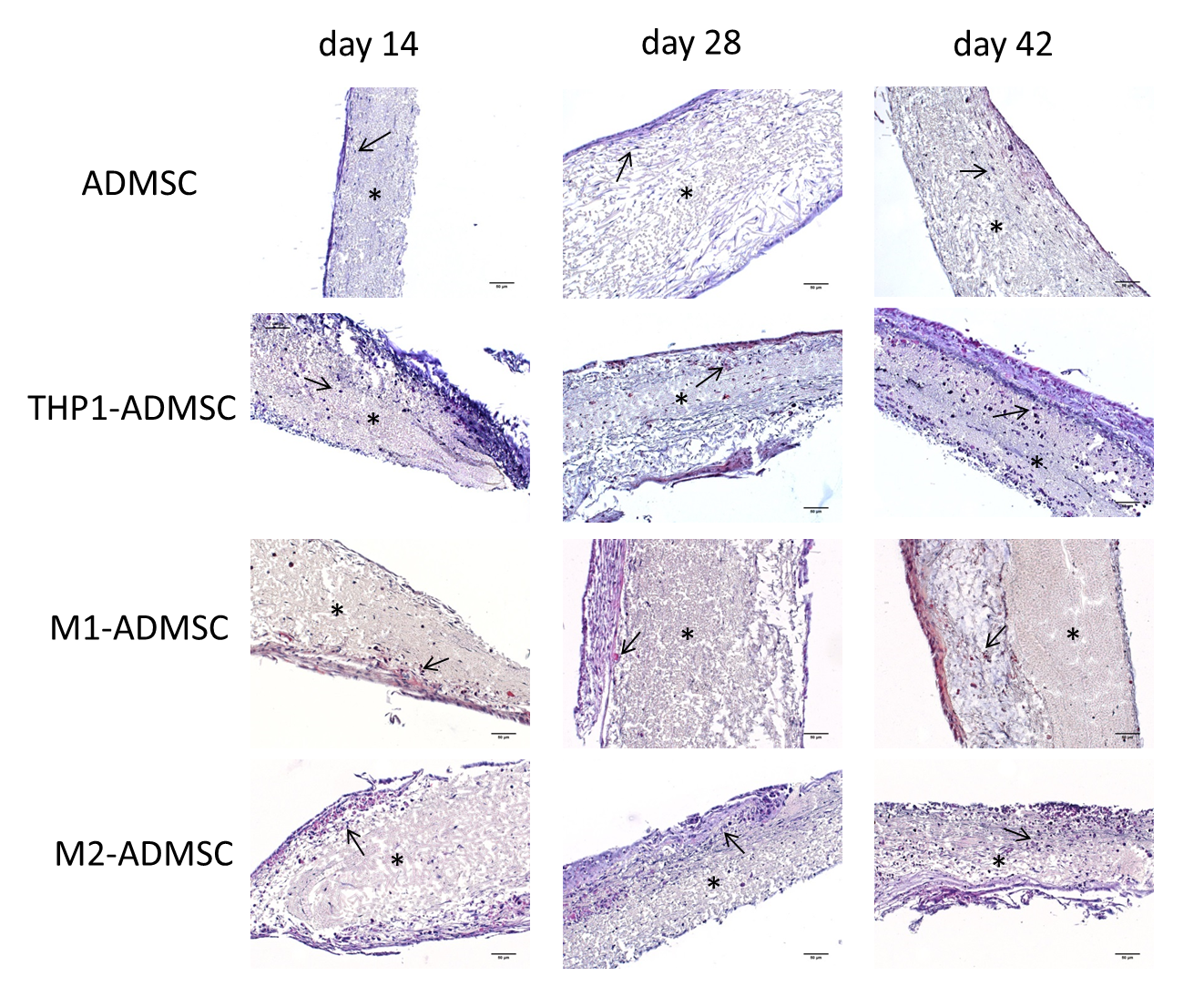

Supplement: Supplementary file 1 — Figure S1. HE‐staining of paraffin sections for each experimental group after 14, 28 and 42 days of culture. Most cells (shown by arrows head) were distributed on the surface of the scaffolds at day 14. More cells were observed entering the scaffolds from day 14 to day 42. A large number of cells mounted layer upon layer on the surface of the scaffolds. “*” indicates scaffolds. Scale bar, 50μm. [file TERM-13-785-s001.tif]

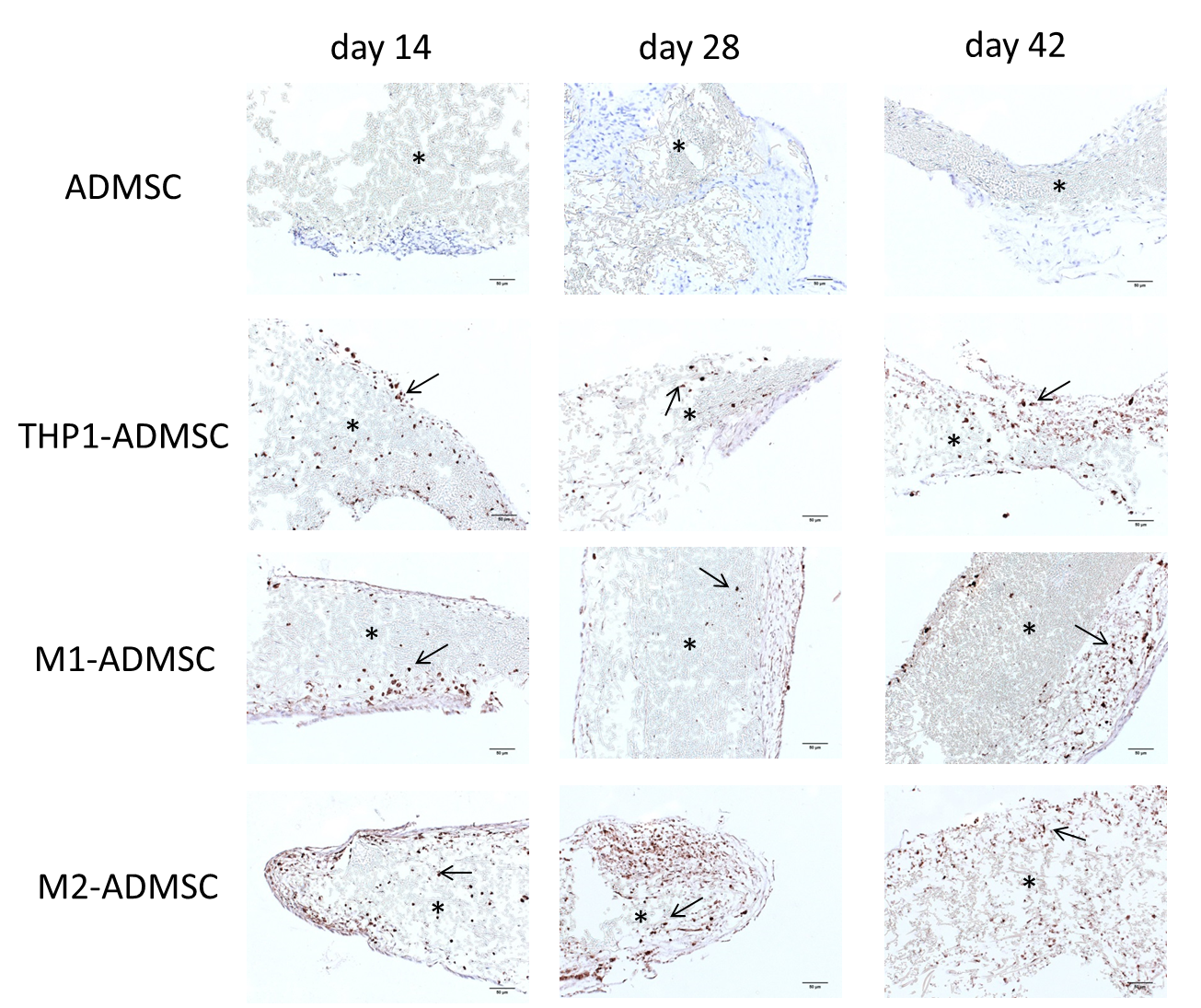

Supplement: Supplementary file 2 — Figure S2. CD68 staining of different group of PLGA/PCL scaffolds after 14, 28 and 42 days of culture. CD68‐positive cells stained in brown (shown by arrows head). No CD68‐positive staining was observed for the ADMSCs mono‐culture. “*” indicates scaffolds. Scale bar, 50μm. [file TERM-13-785-s002.tif]

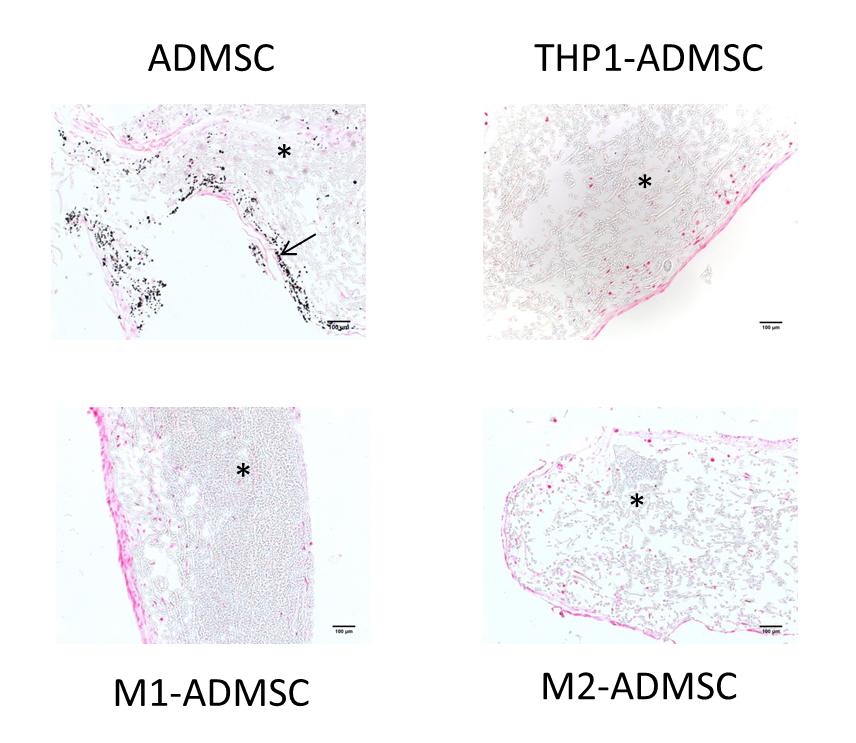

Supplement: Supplementary file 3 — Figure S3. Von Kossa staining of different group of PLGA/PCL scaffolds after 42 days of culture. Von Kossa staining was used to monitor mineral deposition (specifically PO4 3‐) within the scaffolds. Mineral deposition stained in black (shown by arrows head). ADMSCs mono‐culture showed superficial mineralization at day 42. “*” indicates scaffolds. Scale bar, 100μm. [file TERM-13-785-s003.tif]
